# Prevalence and type distribution of high-risk human papillomavirus in patients with cervical cancer: a population-based study

**DOI:** 10.1186/1750-9378-8-20

**Published:** 2013-06-06

**Authors:** Mohammadreza Haghshenas, Tahereh Golini-moghaddam, Alireza Rafiei, Omid Emadeian, Ahmad Shykhpour, G Hossein Ashrafi

**Affiliations:** 1 Molecular and Cell Biology Research Centre, Faculty of Medicine, Mazandaran University of Medical Sciences, Mazandaran, Iran; 2 Faculty of Medicine, Mazandaran University of Medical Sciences, Mazandaran, Iran; 3 SEC Faculty, Penrhyn Road, Kingston University London, Kingston upon Thames, UK KT1 2EE

**Keywords:** Human papillomavirus, Cervical cancer, Genotype of HPV, PCR

## Abstract

**Background:**

Cervical cancer is the greater cause of cancer death in women in many developing countries. Persistent infection with human papilloma virus (HPV), primarily high risk types 16 and 18, is recognized as a causal and essential factor for the development of cervical cancer. We aimed to determine the distribution of high-risk HPV genotypes in archival biopsies with cervical carcinoma in patients from Mazandaran Province, Northern Iran.

**Methods:**

A total of 98 paraffin-embedded cervical samples consisted of 63 Squamous Cell Carcinomas (SCC), 4 Adenocarcinomas, 19 Cervical Interaepithelial Neoplasia grade I (CIN-I), 4 CIN-II and 8 CIN-III diagnosed during 2009–2011, were selected to perform high risk HPV genotyping using AmpliSens^(R)^ HPV HCR DNA genotyping kit. The prevalence of HPV infections was assessed in low and high grade cervical lesions by age.

**Results:**

Of the 98 cervical samples analysed by DNA PCR, 78 (79.59%) were positive for HPV DNA. HPV was detected in the 52 of SCC, 4 of Adenocarcinomas, 14 of CIN-I, 4 of CIN-II, and 4 of CIN-III for HPV. From the 78 HPV positive samples, 23 (29.5%) samples were positive for HPV type 16, 32 (41%) were positive for HPV 18, 19 (24.4%) were positive for HPV 45, and 4 (5.1%) of cervical specimens were positive for HPV 39.

**Conclusions:**

This study provides valuable baseline data for future assessment of the impact of current prophylactic vaccination programs that is protective against the two most common oncogenic types of HPV found in cervical cancer, HPV-16 and HPV-18, but not against other high-risk mucosal HPVs, 39 and 45, reported in this population.

## Background

Cervical cancer is one of the most common types of cancer affecting women worldwide with over 500,000 new cases estimated and over 250,000 deaths each year. The greatest burden of disease is in developing countries, but in spite of better screening and surveillance cervical cancer is still a significant burden also in developed world [1-3]. The risk of cervical cancer has increased in parallel with the incidence of certain genotypes of human Papillomavirus (HPV). Therefore, the presence of these genotypes indicates a significant risk factor for the development of cervical cancer [4-7]. HPVs infect cutaneous and mucosal epithelial cells of the anogenital tract, which can lead to a variety of diseases with a range of severities. The mildest form of HPV disease is low grade intraepithelial neoplasia (CIN-I). These lesions can persist and progress to high grade disease (CIN-III) and invasive cervical cancer [8,9]. HPVs are also found in cancers of the tonsils, anus, penis and cancer of neck [10,11].

High-risk HPV 16 and 18 are found with the highest frequencies in cervical cancer and account for approximately two thirds of all cervical carcinomas worldwide [12,13], with HPV-16 occurring most frequently [14]. It has been demonstrated that the presence of even minimal amounts of HPV DNA is associated with an increased risk in the development of cervical cancer [15]. Considering the broad interest in HPV vaccines, it is very important to verify the prevalence of the various HPV types worldwide, especially the high-risk ones. Despite the medical importance and the high incidence rate of cervical cancer, there is lack of information on the incidence of the HPV genotypes and the provincial differences in their distribution in Iranian population. This study was designed to determine and analyse the distribution of high risk HPV genotypes present in archival biopsies of cervical tumor tissue of patients from Mazandaran province, Northern Iran.

## Methods

### Cervical sample collection

This cross-sectional study, took place during 2009–2011 by approval of the Scientific Ethics Committee of the Mazandaran University of Medical Sciences, Sari. A total of 98 formalin-fixed and paraffin-embedded cervical tissue fragments were retrieved from patients in attendance at Imam khomini Hospital Infections Disease Center, Mazandaran province, Northern Iran. Serial sections (4-7 μm thick) were cut from each specimen. Separate disposable items such as gloves, feather blades and tubes were used to minimise any cross-contamination between samples. The first and last sections were used for histopathological evaluation and intermediate sections were transferred into sterile micro-tube for HPV DNA detection. Additional information was obtained from each patient and collected by means of a questionnaire (age and suspected sources of infection/high-risk sexual relation, for example).

### DNA extraction and HPV DNA detection

Sections of paraffin embedded samples in each tube went through deparaffinization with xylene, and rehydration in graded ethanol. Genomic DNAs from tissue section were isolated using the AccuPrep® Genomic DNA Extraction Kit (Bioneer Corporation, Korea) according to the manufacturer’s guidelines. HPV genotyping was performed using the HPV- HCR Genotype-Eph kit (AmpliSens^(R)^, Russia). The kit is based on simultaneous amplifying in a one tube (multiplex-PCR) of four types of HPV DNA and allows the user to detect infections and co-infections of high risk HPV genotypes; HPV-16, 31, 33, 35, HPV-18, 39, 45, 59 and HPV-52, 56, 58, 66.PCR amplification for detection of HPV twelve types DNA were run in three tubes. HPV-positive and negative samples were included for every sample run.

To determine HPV genotypes the amplified PCR products were run in 1.5% agarose gel stained by ethidium bromide. Since all amplified products had different length, genotypes of the virus were analysed by electrophoresis and visualized by an ultraviolet light trans-illuminator. Bands of appropriate size were identified by comparison with DNA molecular weight markers which are a set of known DNA fragments.

The adequacy of the DNA in each specimen for PCR amplification was determined by the detection of the *β*-globin gene.

## Results

**Figure 1:**
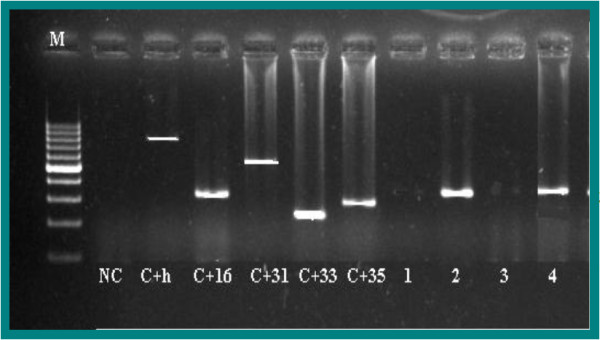
**Gel electrophoresis pattern of high-risk HPV type 16 by genotype specific primers amplification.** M= DNA ladder (100 bp-1000 bp), NC=Negative Control (TE-buffer) (No band), C+h= Internal Control human DNA (723 bp), C+16= Positive Control DNA HPV type 16 (325 bp), C+31= Positive Control DNA HPV type 31 (520 bp), C+33= Positive Control DNA HPV type 33 (227 bp), C+35= Positive Control DNA HPV type 35 (280 bp), 2 and 4= Positive clinical samples (type 16 (325 bp)), 1 and 3= Negative clinical samples.

**Figure 2:**
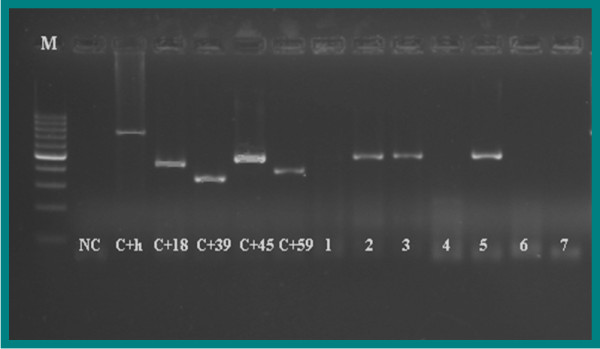
**Gel electrophoresis pattern of high-risk HPV type 45 by genotype specific primers amplification.** M= DNA ladder (100 bp-1000 bp), NC= Negative Control (TE-buffer) (No band), C+h= Positive Control DNA human (723 bp), C+18= Positive Control DNA HPV type 18 (425 bp), C+39= Positive Control DNA HPV type 39 (340 bp), C+45= Positive Control DNA HPV type 45 (475 bp), C+59= Positive Control DNA HPV type 59 (395 bp), 2,3, and 5= Positive clinical samples (type 45 (475 bp)), 1, 4, 6, and 7= Negative clinical samples.

**Figure 3:**
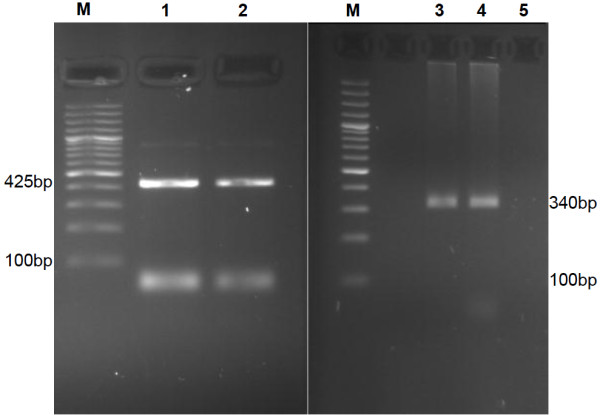
**Gel electrophoresis patterns of high-risk HPV18 and HPV39 genotypes by genotype specific primers amplification.** M= DNA ladder (100 bp), 1 is positive control type 18 (425 bp), 2 is positive clinical samples type18 (425 bp), 3 is positive control type 39 (340 bp), 4 is positive clinical samples type39 (340 bp) and 5 is negative clinical samples.

The results of the 98 patients with an abnormal cervical biopsy diagnosis were collated (Table 1). The mean age of patients was 52.6 ± 13.4 years. All biopsies were pathologically characterised. Pathological examinations showed that out of 98 patients, 63 (64.29%) had Squamous Cell Carcinomas (SCC), 4 (4.08%) had Adenocarcinomas, 19 (19.39%) had Cervical Interaepithelial Neoplasia grade I (CIN-I), 4 (4.08%) had CIN-II and 8 (8.16%) of patients had Cervical CIN-III. HPV-DNA was detected in 78 of 98 patients (79.59%) with cervical specimens using PCR (Table 1). The resulting fragment of high-risk HPV genotypes 16, 18, 39 and 45 were clarified effectively using agarose gel electrophoresis. As a standard procedure, positive and negative controls were included for every sample run (Figures 1, 2 and 3). The most common HPV genotypes detected in this study were type 18 followed by type 16, 45, and 39. HPV type 16 was present in 23 (29.5%) of cervical specimens, HPV-18 in 32 (41%) of cervical specimens, HPV-45 in 19 (24.4%) of cervical specimens and HPV-39 in 4 (5.1%) of cervical specimens.

**Table 1:** HPV genotype distribution in women with cervical abnormalities

**Pathological status**	**HPV genotype**				**Total HPV positive**
	**16**	**18**	**39**	**45**	
SCC	17 (32.69%)	18 (34.62%)	4 (7.69%)	13 (25%)	52
Adenocarcinomas	2 (50%)	2 (50%)	-	-	4
CIN1	4 (28.58%)	8 (57.14%)	-	2 (14.28%)	14
CIN2	-	4 (100%)	-	-	4
CIN3	-	-	-	4 (100%)	4
Total	23 (29.49%)	32 (41.03%)	4 (5.13%)	19 (24.36%)	78

Our results demonstrated that the most prevalent HPV genotypes in SCC specimens were HPV-16 and 18, Adenocarcinoma specimens displayed HPV-16 and 18, CIN-I specimens displayed HPV types 18, 16, and 45. CIN-II specimens were found to be positive for HPV-18 and CIN-III specimens produced HPV-45 (Table 1).

The HPV genotype distribution by age demonstrated that HPV 16 and HPV 18 were found in all age range of patients with abnormal cervical biopsies, while that was not the case for HPV 45 and HPV 39. HPV 45 was the most prevalent in the 60–69 year age group and was not found in 20–29 and 70–79 year group respectively. HPV 39 was found in 40–49 and 60–69 years no other age groups (Table 2).

**Table 2:** Distribution of HPV genotype according to age group of patient

**Age groups**	**HPV genotype**				**Total HPV positive**
	**45**	**18**	**39**	**16**	
20-29	2 (50%)	2 (50%)	-	-	4
30-39	2 (25%)	4 (50%)	-	2 (25%)	8
40-49	4 (19.05%)	11 (52.38%)	2 (9.52%)	4 (19.05%)	21
50-59	7 (35%)	9 (45%)	-	4 (20%)	20
60-69	2 (13.33%)	2 (13.33%)	2 (13.33%)	9 (60%)	15
70-79	6 (60%)	4 (40%)	-	-	10
Total	23 (29.49%)	32 (41.03%)	4 (5.13%)	19 (24.36%)	78

## Discussion

Given the well-established strong relationship between cervical neoplasia and HPV infection, a high frequency of oncogenic HPV-DNA in cytologically abnormal biopsies is anticipated. Infection by certain types of human papillomaviruses is recognised as a causal and necessary factor for cervical cancer [8,9]. Cervical cancer represents the second most common malignancy among women around the world and contributes to 9·8% of all female cancers [1,2].

Histopathological results on 98 cervical biopsies of our study, revealed that 63 (44%) of abnormal cervical specimens were SCC, 4 (4.1%) were adenocarcinoma, 19 (19.4%) were CIN-I, 4 (4.1%) were CIN-II and 8 (8.2%) were CIN-III. The results of high-risk HPV typing on these samples showed that HPV-DNA was found in 56 (83.5%) of cervical carcinoma specimens. This was reported at a much higher prevalence in Italy (99.3%) [16], Haifa (98.5%) [17], Norway (92%) [7], Lithuania (92%) [18], Australia (90%) [19], India (87.8%) [20], and at an almost similar level in and China (75-83%) [21]. However a study from Turkey found a much lower HPV prevalence of 36% [22].

The main HPV types detected in our study are HPV 18 in 32 (41%), HPV 16 in 23 (29.5%), HPV 45 in 19 (24.4%) and HPV 39 in 4 (5.1%) of the HPV positive cases. Worldwide, approximately 70% of women diagnosed with cervical cancer are found to carry HPV type16 and HPV type18 [23,24]. This relates positively to our overall detection of HPV type 16 (28.35%) and 18 (29.85%) in HPV positive cases of patients with cervical carcinomas.

The geographical variations in HPV distributions may be due to the difference in the number of samples in each study and also the cultural attributes in some countries. Establishment of distinctive religious and cultural limitations on having multiple sexual partners in countries like Iran can prevent people from being involved in risky sexual contacts, which consequently can lower the risk of HPV infection. On the other hand, limited awareness of Iranian women with respect to HPV and its implication in cervical cancer aetiology, and also lack of educational programmes on HPV can be the reasons for 79.59% of HPV genital infection reported in this study.

Previous investigations in different parts of Iran [25-27] demonstrated HPV type 16 as the main oncogenic type of HPV associated with cervical cancer while HPV types 18, 33, 45, 31 and 52 were reported at lower percentage in patients with cervical cancer.

This is in contrast with our findings that highlight HPV type 18 (29.85%) and HPV types 16 (28.35%) as the most prevalent type detected from HPV positive cases and HPV types 45 (19.4%) and 39 (6%) being found to a lesser extent in our cervical carcinoma group. This may be attributable to geographical variation in different parts of Iran that needs to be investigated further.

In comparison to previous studies in Iran, this study has provided more detailed information on the pattern of the relative prevalence of high risk HPV infections in low and high grade cervical lesions by age. HPV types 16 and 18 were found in all age groups. This can be a characteristic feature of the population where HPV transmission continues into middle age and may contribute to cervical cancer incidence. Women in this population may also have decreased ability to clear HPV infections, possibly due to age-related reduced immune responses.

Our results also showed that the prevalence of HPVs were peaked among women with SCC and fell with decreasing grade lesions to CINs. This observation is consistent with other reports, which highlighted the highest HPV positivity rate in SCC samples, but lower rates of HPV prevalence in other histological classifications [28,29].

Even though a significant HPV positivity was expected in all cervical samples, our results show there was a contrast in the percentage of HPV detected CIN3 (50%) and SCC (82.5%) compared to other cervical sample types, where the HPV detection rate was 100%. There may be a number of reasons for this, such as sample size and the time length individual samples may have been stored in paraffin before testing occurred. To account for any differences in the sample storage, extremely sensitive HPV detection methods were administered and therefore the results are reflective of what was found using these sensitive methods.

In summary, an important strength of this study is that in addition to detection of the most common types of cervical cancer related HPV (type 16 and 18), we have detected other high risk types of HPV DNA, 39 and 45, in samples collected from women with cervical lesions. This, in line with other studies on the prevalence of HPV genotypes in cervical cancer, provides a baseline to assess the impact of current HPV vaccination.

## Conclusions

The presence of HPV genotypes 16 and 18 in 58.2% cervical cancer in this study will strongly support the implementation of current vaccination against HPV infection (aimed to prevent HPV 16 and 18) in north part of Iran. However, since the protection induced by the current vaccines is predominately type specific, the identification of HPV45 and HPV 39 genotypes in this study can be good evidence for generating a new broad spectrum prophylactic vaccine that can prevent greater than 56% of cervical cancers.

There may still be other genotypes of HPV that can be detected in different geographical areas. Therefore, further investigation on HPV genotyping in the wider population would be required.

## Competing interests

The authors report no competing of interests.

## Authors’ contributions

MH and GHA were responsible for the overall planning of the study. TGM was responsible for sample collections from the patients. AR participated in study design and helped revise the manuscript. OE carried out the histopathology evaluation of the samples. AH carried out DNA extraction and PCR analysis. MH drafted the manuscript; GHA interpreted results, revised and finalised the writing of the manuscript. All authors read and approved the final manuscript.
